# The Hollow Square: A Rejoinder

**Published:** 1918-12-28

**Authors:** 


### IDEALS IN TOWN-PLANNING.

### The Hollow Square: A Rejoinder.

By A SOCIAL WORKER.

The question of the hollow square as a principle of building reconstruction in our towns grows in interest. At first sight, no doubt, such a plan seems Utopian, or at best impossibly idealistic. But great changes are needed, and unless we have some ideal to work towards we may plough towards a grazing cow. Moreover, a little observation shows that some opportunities for municipal experiment are fast coming within reach in consequence of changes made by the war, and that unless utilised they may only tend to the manufacture of new slums.

The shifting of fashion has, of course, always caused districts of towns to deteriorate. People leave a handsome house for one in a more approved neighbourhood, and of late the breaking-up of homes by death, and the inability or disinclination of the heir to keep up a family house, also the difficulties of the servant question, have multiplied such cases immensely. The landlord, who is sooner or later obliged to accept a much-lowered rent, does up the building as cheaply as possible, avoiding structural repairs and improvements, and if eventually the house is occupied by a number of poor families the conditions are wholly unsuited to healthy living. In London the results of this process may be seen in many places. The grandfather of the present writer had his town house in Newgate Street, before fashion urged that a garden, severely railed in and screened from view by close-cropped privet, should interpose between a man's front windows and those of his neighbour. But such fine squares as Milner Square, Islington, have suffered change, and, though to mention them would be invidious, there are districts highly fashionable yesterday where ominous ranges of house agents' boards suggest that a like fate awaits the tall mansions, stately in appearance, but inconvenient in practical management, which are described as "desirable family residences."

The Square gardens are sombre places enough in fashionable quarters where the railings are kept painted and the privet clipped, and perhaps even a few geraniums are bedded out in summer. The best that can be said of them is that they serve as air-spaces, and nurses and children sometimes gossip and play there. A few have tennis courts, not in great demand, dogs are allowed runs on the grass, and birds more interesting than the ubiquitous sparrow occasionally nest there in despite of cats. But imagine railings and privet replaced by posts and chains, and the pleasant sward open to view—

"Why, of course, all the little boys and girls from the mews would pour in and——"

Precisely, and all the surrounding children of the poor who now play in back streets or are laboriously dragged impossible distances by elder brother and sister to park or Tube station would follow them. Standing-room, in fact, would be hardly available on a fine afternoon. But supposing such a square to be taken over by the local authority, who would begin by adapting the houses—as large houses taken for hospitals are every day adapted—to healthy living for several families, the second step might be to provide an entrance to the other side of the houses. Such entrances do, of course, often exist already. The garden would then be available for all the inhabitants, the roadway being taken into it and the exits closed. The pavement would remain as a stone terrace, usefully dry and flat, and it might be provided with seats. This abolition of the roadway would eventually be a great gain. Experiments made before the war have shown how serious a danger to health lies in the constant breathing in of road dust, and still more of the dust of crowded streets, at that time rendered especially harmful by the constant passing of motors and steam lorries. According to experiments carried out in Paris by M. M. Sartory and Langlais, in 1911, air taken from a street near the Madeleine had at 8 A.M. 545 bacteria per cm., at 5 P.M., 20,800. Air from the Place de la Concorde had at 8 A.M. 640 bacteria, at 2 P.M. 72,000, and at 7 P.M. 88,000. Where a house was being pulled down air which would normally contain 500 to 10,000 bacteria per cm., showed 112,000 to 240,000. From this and many more dangers children who now habitually play in the street would be defended by the plan of the hollow square. In the garden would be a play shelter with roof, but with a wall only on the north side, and here, of course, might well be the crèche and nursery school in one. The four spaces left at the former exits would be useful sites for extra building—common kitchen and restaurant, bath-house, club-rooms.

If the idea seems beyond the reach of practical politics, let one or two things be remembered. First, that large changes will have to be made to bring popular housing anywhere near our minimum idea of health and decency. Secondly, that these will not, *cannot*, be left entirely to private adventure. Municipal housing, with municipal control, will certainly develop rapidly, but by utilising adapted buildings at present standing, vast outlay on bricks and mortar would be saved. Finally, that unless we take stringent measures before long, our descendants a few generations hence are likely to be as amazed at the town conditions in which we were willing to live as we ourselves are at the customs of the Stone Age, or, much later, at those of London before the Plague and Fire.

